# Thyroid Storm in Lupus: A Rare Cause of Unrelenting Pyrexia

**DOI:** 10.31138/mjr.34.1.101

**Published:** 2023-03-31

**Authors:** Varsha R. Bhatt, Sanjay M. Khaladkar, Manaswini Edara

**Affiliations:** 1 Department of Medicine, Rheumatology Clinic, Dr. D.Y Patil Medical College, Hospital and Research Centre, Pune, Maharashtra, India,; 2 Department of Radiodiagnosis, Dr. D.Y Patil Medical College, Hospital and Research Centre, Pune, Maharashtra, India,; 3 Department of Medicine, Dr. D.Y Patil Medical College, Hospital and Research Centre, Pune, Maharashtra, India

**Keywords:** lupus, fever, thyroid storm

## Abstract

Systemic lupus erythematosus may present with fever, and it is a challenge to attribute fever to a particular cause. Very rarely it can be due to hyperthyroidism. Thyroid storm is a medical emergency causing unrelenting pyrexia. Here we report a case of a young female who first presented as fever of unknown origin (FUO), was subsequently diagnosed as neuropsychiatric lupus, and in whom the cause of unrelenting high fever, which did not respond to adequate immunosuppression to quell disease activity, was documented to be thyroid storm after excluding all other causes such as infection and malignancy. To our knowledge, this is the first case of this kind reported in literature, although cases of thyrotoxicosis preceding or following the diagnosis of lupus is known. Her fever resolved after starting antithyroid drugs and beta blockers.

## INTRODUCTION

Fever is a common manifestation of lupus.^1^It occurs in 36–86% patients, mostly due to lupus disease activity, infection, malignancy, drug reactions and theoretically some rare aetiologies like thyrotoxicosis.^2^ It is a challenge to attribute fever in lupus to a particular.^3^ Due to multiple autoantibody production, various organs are involved, including the thyroid gland. Clustering of multiple autoimmune diseases has been reported by some studies.^4^ Increased frequency of thyroid autoimmunity was reported among lupus, rheumatoid arthritis, systemic sclerosis, and mixed connective tissue disease (MCTD) patients.^5^

According to literature, cumulative incidence of thyroid diseases in lupus patients was 17.5%, including hyperthyroidism 6.4%, hypothyroidism 8.5% and autoimmune thyroid disease 5.4%.^4,6^

Thyroid storm is an acute manifestation of thyrotoxicosis, often precipitated by a physiologically stressful event. It can be diagnosed using the Burch-Wartofsky Point Scale (BWPS). If unrecognized or left untreated, it may result in cardiovascular collapse and death. It often presents as high-grade pyrexia requiring emergent treatment.^7^ These symptoms may not be attributed correctly in cases of high lupus activity and hence diagnosis is often missed. But thyroid storm occurring in course of a highly active lupus is rare.^8^

Here we report a case of a young female who first presented as fever of unknown origin (FUO), was subsequently diagnosed as neuropsychiatric lupus, and in whom the cause of unrelenting high fever, which did not respond to adequate immunosuppression to quell disease activity, was documented to be thyroid storm after excluding all other causes such as infection and malignancy. To our knowledge, this is the first case of this kind reported in literature, although cases of thyrotoxicosis preceding or following the diagnosis of lupus is known.

## CASE DESCRIPTION

A 26-year-old female presented to this hospital with high grade continuous fever, and significant weight loss for six weeks. She also reported pathological hair loss and inflammatory joint pains involving small joints of hand for the last three months. She had no history of cough, abdominal pain, dysuria, diarrhoea, bleeding from any site, bone pain, headache, or chills. She had no history of photosensitivity, rashes, ulcers, Raynaud’s phenomenon, myalgias, muscle weakness, skin tightening or pregnancy morbidities. On examination her temperature was 40 °C (104 °F), which initially responded mildly to antipyretics. She had synovitis in three metacarpophalangeal and proximal interphalangeal joints of both hands and non-scarring alopecia. In three days of hospitalisation, during her moderately febrile period, she developed generalised tonic clonic seizures and altered sensorium, not limited to postictal phase. She was extensively worked up. She had leukopenia with counts of 3000/cmm, and mild thrombocytopenia. Her liver and renal function tests were normal, and urine showed no albuminuria and cells. Peripheral smear was negative for parasite and rapid malaria test was negative. Dengue, HIV, hepatitis B, and C serologies were negative. Her blood and urine cultures taken thrice were negative. Her chest X ray, abdominal ultrasound, 2 D Echocardiography, chest and abdomino-pelvic Computed Tomography (CT) scans were normal. Her fundus and plain CT head was normal. A lumbar puncture was done which showed normal biochemistry and cytology. The adenosine deaminase level was normal and cerebrospinal fluid (CSF) was negative for gram stains and culture, culture for acid fast bacilli was also subsequently negative. Her CSF PCR for tuberculosis, Herpes Simplex Virus and Japanese Encephalitis were negative. Test for oligoclonal bands in CSF was negative. Her bone marrow aspiration was normal, and culture was negative.

**Figure 1.**
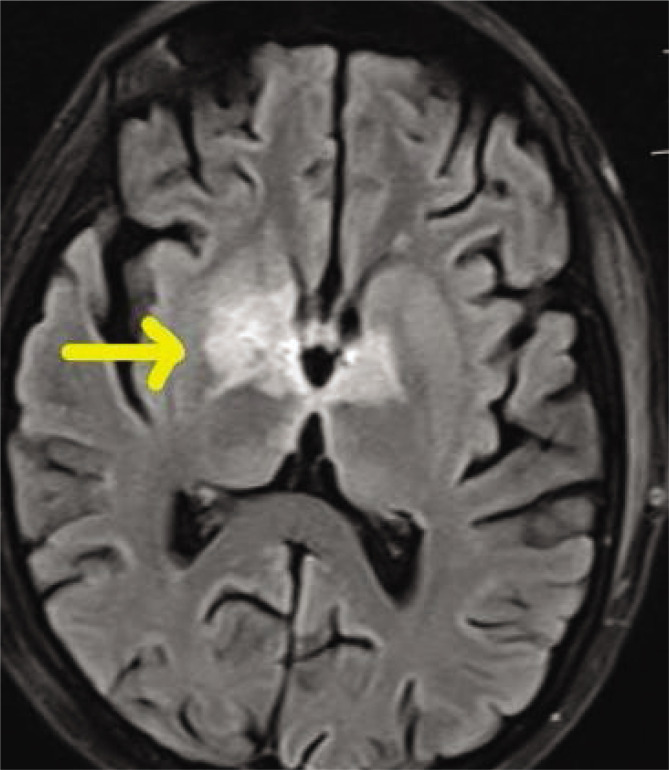
Diffusion weighted image showing restricted diffusion in bilateral basal ganglia, thalami, and internal capsule areas suggestive of acute infarcts.

**Figure 2.**
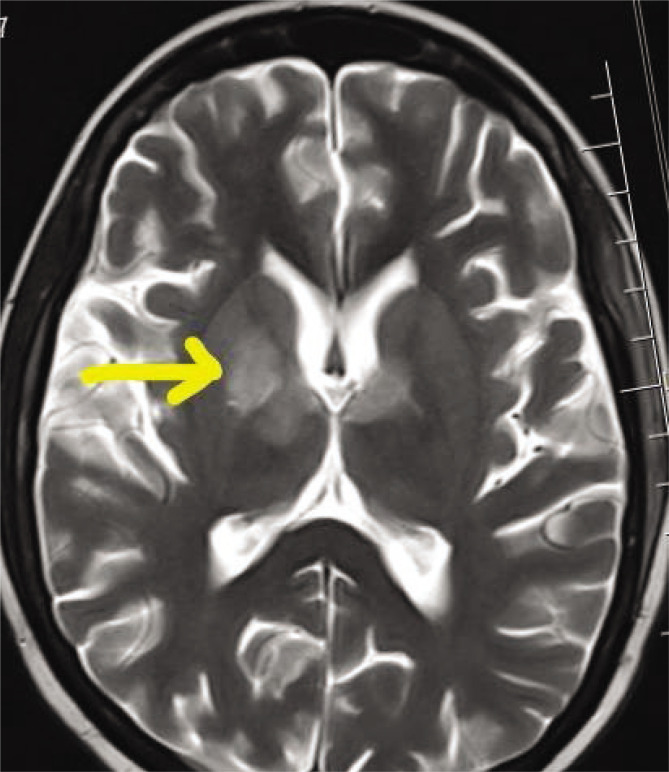
Axial T2 weighted images and FLAIR showing ill-defined hyperintense lesions in bilateral basal ganglia, thalami, and internal capsule.

Antinuclear antibodies were strongly positive by immunofluorescence; 4+ speckled with positive Smith (Sm), Ro-52 and ribonucleoprotein (RNP), but a negative double stranded DNA. She had low complement levels. Her MRI brain showed bilateral acute to subacute infarcts in thalami, basal ganglia and internal capsule, suggestive of small vessel vasculitis. (**Figure 1** and **Figure 2**) Her antiphospholipid antibodies were negative. She was hence diagnosed as case of lupus with neuropsychiatric features with SLEDAI 2K score of 19 points and was given pulse methylprednisolone 1g once a day for three days along with aspirin 150mg and clopidogrel 75mg with atorvastatin 40mg daily. She was also given first pulse of cyclophosphamide 750mg/m^2^ intravenously as per National Institute of Health (NIH) protocol. She initially stabilised with no seizures and was conscious but her fever, although reduced in intensity, never resolved. She subsequently developed hyperpyrexia with temperature of 41.1 °C (106 °F). She had tachycardia of 130–140 beats/min and developed diarrhoea. Her blood and urine cultures were consistently negative, total leukocyte counts had normalised and she had normal procalcitonin. Her thyroid function tests revealed a very low thyroid stimulating hormone (TSH) of <0.002 mIU/L, T3 of 360 ng/ml and T4 of 16 mcg/ml with positive thyroperoxidase antibody and thyroglobulin antibody. Hence the diagnosis of thyroid storm was made according to the Burch-Wartofsky Point Scale (BWPS) for diagnosis of thyroid storm.

She was treated with carbimazole 40 mg 8 hourly through Ryles tube, and Propranolol 60mg 6 hourly. With this her fever miraculously abated and heart rate normalised. Unfortunately, her lupus activity was high, and she subsequently developed generalised seizures again which required ventilation. Her cytopenia had worsened and she had albuminuria. Her SLEDAI 2K score increased to 23 points. Plasmapheresis was started. She improved but subsequently developed ventilator associated pneumonia and sepsis. Her TSH had increased to 0.2 mIU/L. She succumbed to severe sepsis.

## DISCUSSION

Pyrexia can be a presenting feature of lupus and can occur at all stages of the disease. It is a challenge to diagnose and attribute it to either disease activity or infection, a combination of the two and rarely malignancies, hemophagocytic syndrome or coexisting thyrotoxicosis.^2^ Although thyroid diseases can occur with lupus, thyrotoxicosis is rare. In a cohort study, the overall rate of hyperthyroidism in the lupus group was 6.4%.^4^

The frequency of autoimmune thyroid disease (AITD) among 189 lupus patients was 6.3%, with 2.6% in the hyperthyroid group and 3.7% in the hypothyroid group.^9^ In yet another study, a hundred lupus patients were also evaluated for AITD, in comparison with 100 age- and sex-matched controls. Thyroid dysfunction was reported in 36 (36%) lupus patients (14 [14%] with clinical hypothyroidism, 2 [2%] with subclinical hyperthyroidism, and 12 [12%] with subclinical hypothyroidism). Prevalence of thyroglobulin antibody in lupus patients was 30%. None of these studies reported occurrence of thyroid storm.^10^

Thyroid storm is a medical emergency. Acute heart failure may be the initial event and later, respiratory failure, disseminated intravascular coagulation (DIC), gastrointestinal signs, neurological collapse and sepsis may occur. To reduce mortality and to improve survival rate early suspicion, prompt diagnosis and intensive treatment are essential. Burch-Wartofsky Point Scale (BWPS) for diagnosis of thyroid storm, is an empirically derived scoring system, which includes severities of symptoms of multiple organ decompensation (thermoregulatory dysfunction, tachycardia/atrial fibrillation, disturbances of consciousness, congestive heart failure and gastrohepatic dysfunction). The cardinal feature of thyroid storm involves the Central Nervous System (CNS) with restlessness, delirium, psychosis, seizures, and a change of mental status. If CNS features are present, the presence of only 1 of the following conditions makes the diagnosis: fever >100.4F (38 degree C), increased heart rate >130, severe congestive heart failure or a gastrointestinal issue (diarrhoea, nausea/vomiting). If patient did not have CNS features then they had to have three of the above conditions. In this patient, CNS manifestation was clinically attributed to lupus, but she fulfilled the other criteria of thyroid storm, namely fever, tachycardia and diarrhoea and the score was >45 according to Burch-Wartofsky Point Scale (BWPS) for thyrotoxicosis. The fever resolved completely after starting antithyroid drugs.^7^

## CONCLUSION

This patient was not a known case of lupus, presenting for the first time with a pyrexia and developed neurolupus; the cause of her unrelenting high-grade pyrexia was proven to be thyrotoxicosis causing thyroid storm. She eventually succumbed to sepsis and her disease, although became afebrile after starting antithyroid medication. The challenge was the diagnosis of pyrexia in her, and attributing it to thyroid storm while her neuropsychiatric manifestations were confirmed to be due to lupus with a high disease activity.

Although rare, thyrotoxicosis must be kept in mind as a cause of unrelenting fevers in a lupus patient, because if left untreated it can lead to thyroid storm and may be fatal.
